# Post-translational modifications of protein and lung cancer

**DOI:** 10.3389/fonc.2025.1667200

**Published:** 2025-11-11

**Authors:** Ying Zhao, Xiaoyu Song, WenFeng Luo, Fangmei Xie, Jian Shen, JinHua He, Zeping Han, Jinju Huang

**Affiliations:** 1 Central Laboratory, The Affiliated Panyu Central Hospital, Guangzhou Medical University, Guangzhou, Guangdong, China; 2 Geriatric Medicine Institute of Panyu District, The Affiliated Panyu Central Hospital, Guangzhou Medical University, Guangzhou, Guangdong, China; 3 Rehabilitation Medicine Institute of Panyu District, The Affiliated Panyu Central Hospital, Guangzhou Medical University, Guangzhou, Guangdong, China; 4 Intensive Care Unit Ward 1, The Affiliated Panyu Central Hospital, Guangzhou Medical University, Guangzhou, Guangdong, China

**Keywords:** post-translational modifications, lung cancer, diagnosis, treatment, prognosis, progression

## Abstract

Post-translational modifications (PTMs) represent a pivotal regulatory mechanism in cellular processes, wherein the addition or removal of specific functional groups to amino acid residues dynamically modulates protein activity, subcellular localization, expression levels, and interactions with other biomolecules. Key PTMs, including phosphorylation, acetylation, methylation, glycosylation, ubiquitination, and emerging types like succinylation and crotonylation, exponentially diversify the proteome’s functional landscape. In lung cancer, PTMs orchestrate critical pathological processes, such as EGFR phosphorylation-driven proliferation, H3K27me3-mediated epigenetic silencing, and KEAP1 succinylation-regulated redox homeostasis. Recent advances in mass spectrometry (MS), phosphoproteomics, and epigenomic profiling have enabled systematic mapping of PTM networks, revealing their potential as diagnostic biomarkers, therapeutic targets, and predictors of drug response. This review synthesizes the mechanistic roles of PTMs in lung cancer pathogenesis and their translational applications, highlighting multi-omics integration and PTM-targeted therapies as future frontiers in precision oncology.

## Introduction

Primary bronchopulmonary carcinoma, commonly referred to as lung cancer, represents one of the most prevalent and lethal malignancies worldwide, including in China (1). Epidemiological data from 2022 revealed that lung cancer constituted 18.06% of all newly diagnosed malignant tumors in China, ranking as the most frequently occurring cancer. Furthermore, it accounted for 23.9% of total cancer-related mortality, maintaining its position as the leading cause of cancer deaths (2). The insidious nature of early-stage lung cancer often results in asymptomatic progression, with the majority of cases being diagnosed at advanced stages upon clinical presentation. Notably, the overall 5-year survival rate for advanced lung cancer patients remains dismal at approximately 20% (3). Consequently, deciphering the molecular pathogenesis of lung cancer and identifying novel therapeutic targets to enhance patient survival carry profound clinical and scientific implications.

PTMs represent essential biochemical processes involving covalent alterations of amino acid residues that occur co- or post-translationally. These modifications, mediated by specialized enzymatic machinery, dynamically modulate protein structure and function, thereby regulating stability, subcellular localization, and molecular interactions (4). Current estimates suggest that more than 5% of the human proteome comprises enzymes capable of catalyzing over 200 distinct PTM types, including kinases, phosphatases, transferases, ligases, and proteases (5). PTMs can occur throughout the protein lifecycle, with many proteins undergoing combinatorial modifications through sequential proteolytic processing and functional group additions during maturation and activation (6, 7). The expanding repertoire of >400 documented PTM types has dramatically enhanced proteomic complexity and functional diversity (8). Key PTM classes, encompassing phosphorylation, glycosylation, acetylation, methylation, ubiquitination, SUMOylation, succinylation, and crotonylation, regulate fundamental hallmarks of cancer through their unique structural and biochemical characteristics. This review synthesizes their specific roles in lung cancer, providing a systematic analysis of their applications in diagnostics and targeted therapy, alongside their prognostic value (**Table 1**).

**Table 1:** Functional landscape of major PTMs in lung cancer pathogenesis.

PTM type	Key targets	Biological function in lung cancer	Reference
Glycosylation	Cathepsin V, PTX3, N-glycans, TIM-4, GPNMB-EGFR, Galectin-3, SOD, ITGA5, SMAD4/TGF-β signaling, NECTIN2-TIGIT, MUC5AC-VCAN-V1	Promotes immune evasion, metastasis, stemness, diagnostic utility, therapeutic target and therapy resistance	(9–25)
Phosphorylation	EGFR, HER3, LKB1, PI3K/AKT, Bcl-2, p38-MAPK, PI3K/AKT,​p53, ERK5, AKT/GSK-3β, PDHA1, PLK1, Nrf2, SHP1, EGFR-TRAF4-MEKK3-ERK5	Drives proliferation, survival, drug resistance, metabolic reprogramming, immune evasion	(26–44)
Ubiquitination	PKC/FAK, Mxi1, Akt, Smad4, FOXO1/Wnt/β-catenin, Mcl-1, ZEB1, CPNE1, Notch1, Nrf2, Mutant p53, c-Myc, Snail, IGF2BP1, IRAK1/AKR1B10, HSC70	Regulating carcinogenic signals, tumor-suppressor stabilization/inactivation, targeted therapy resistance,oncogene degradation, and metastasi	(47–64)
Methylation	RASSF1, ATIC, SHOX2, PRKCDBP, SOX1, SPAG6, circTFF1/miR-29c-3p/DNMT3A/BCL6 Axis, HOXC-AS3, METTL14, METTL3, SETD1A, SETDB1,SFRP1, hY4F, EGR1/ALDH3A1	Tumor progression, metastasis, immune response, ferroptosis, and epigenetic therapy resistance	(66–80)
Acetylation	ACSS2, MCL1, KPNA2, CCS, GLS, p53, K185/K201, FABP4	Regulates chromatin remodeling, metabolic adaptation, metastasis, and therapy resistance, apoptosis resistance, and immune evasion	(84–93)
Succinylation	SOD1, SUCLG2	Modulates mitochondrial metabolism, redox homeostasis, and tumor proliferation	(96, 97)
Crotonylation	BEX2	Regulates chemosensitivity, mitophagy, and apoptosis evasion	(99)
SUMOylation	Slug, SMAD4, SIRT1, HIF-1α, NRF2, VEGFR2, GATA2, NDRG2, circTLCD4-RWDD3	Influences transcriptional regulation, oxidative stress response, angiogenesis, and drug sensitivity	(101–111)

## Literature search strategy

**Figure 1:**
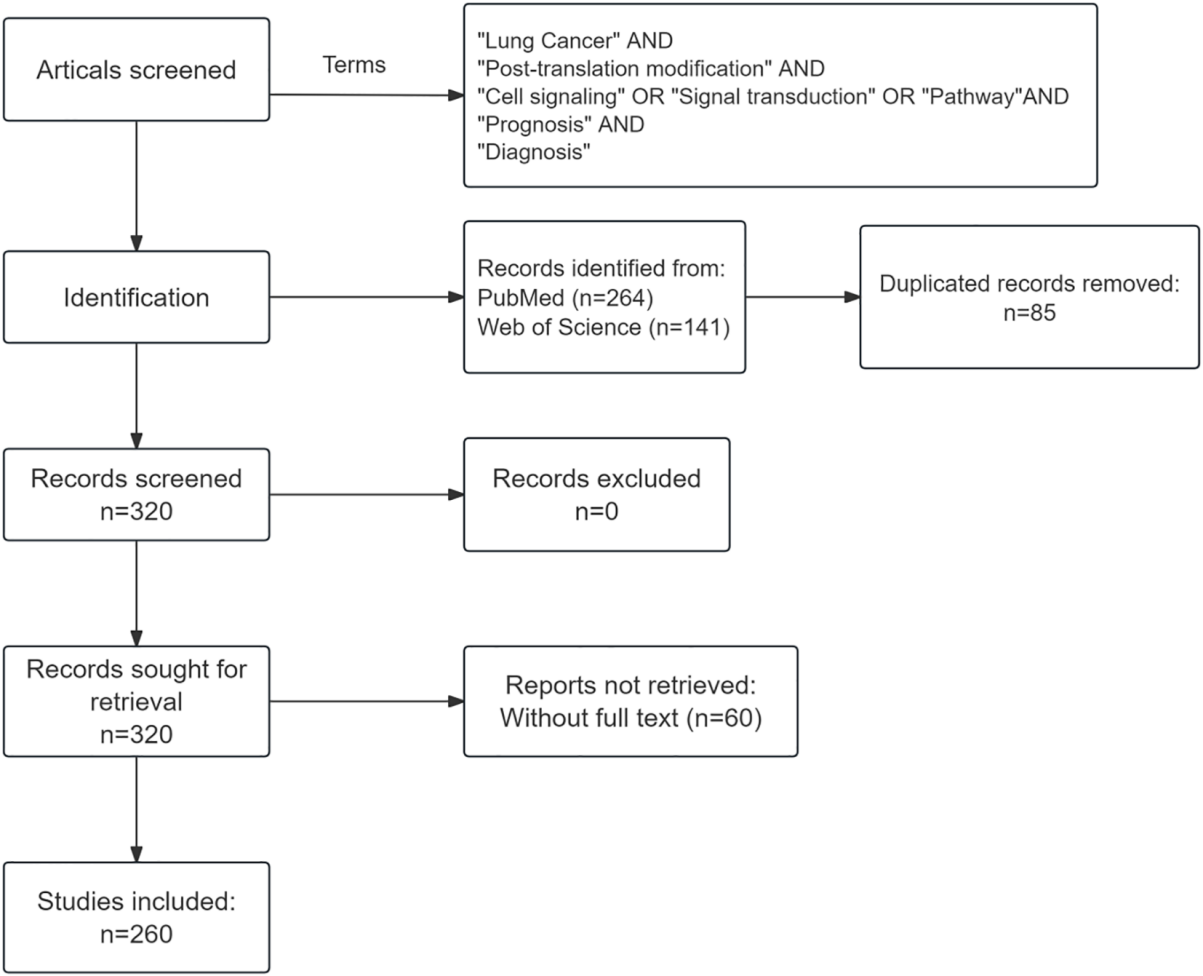
Flow diagram of the article-selection process.

Searches were performed on PubMed and Web of Science using the following key terms: “post-translational modification”, “lung cancer”, “cell signaling”, “signal transduction”, “pathway”, “prognosis” and “diagnosis”, covering publications from 2015 to 2025. The search was limited to articles published in English. The initial search yielded 264 records from PubMed and 141 from Web of Science. After removing 85 duplicates and 60 articles with inaccessible full texts, a total of 260 publications were included in this review (**Figure 1**).

## Glycosylation and lung cancer

**Figure 2:**
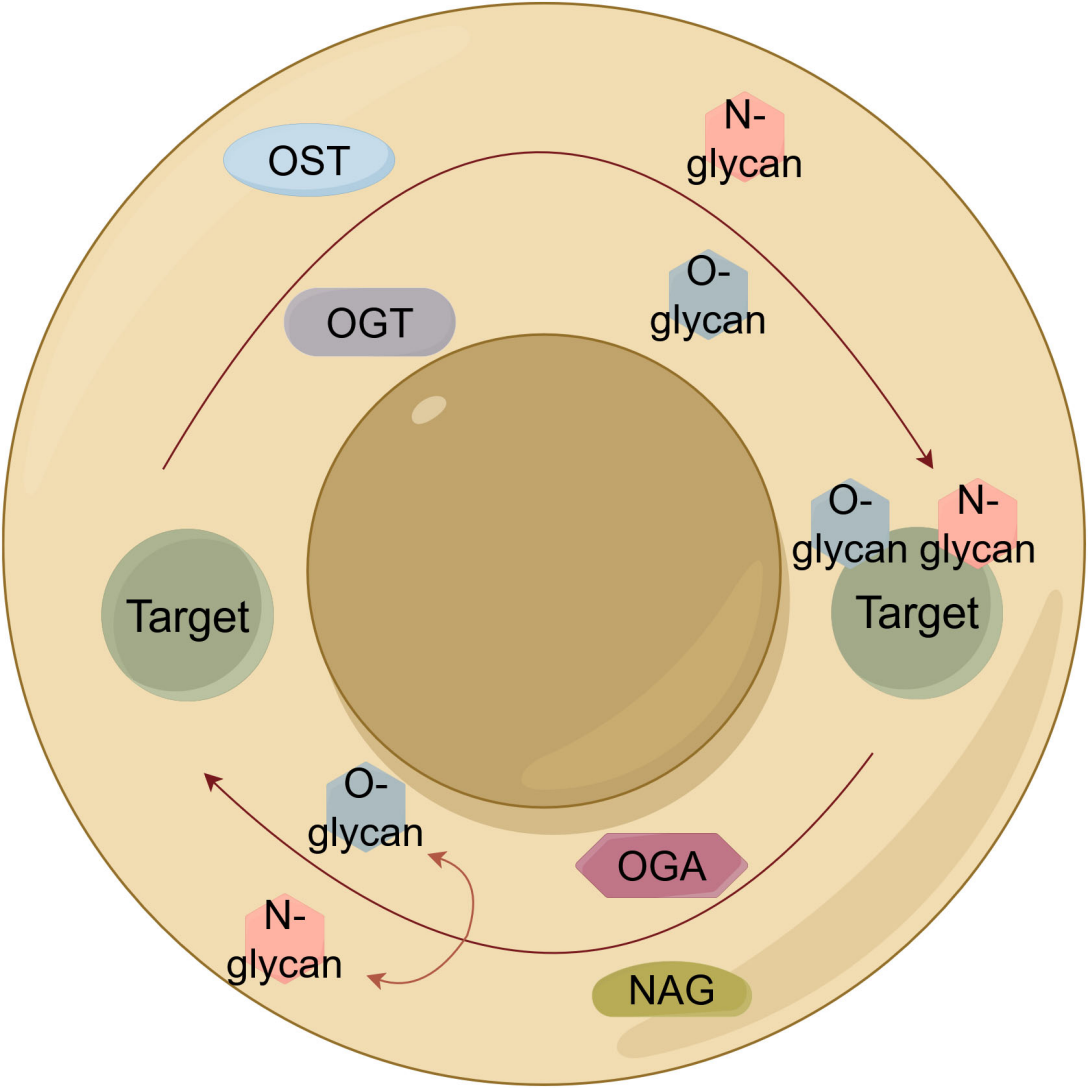
Schematic illustration of protein glycosylation mechanisms in lung cancer. Core pathways of protein glycosylation: N-linked (OST-mediated) and O-GlcNAcylation (OGT/OGA cycle).​ OST, Oligosaccharyltransferase; OGT, O-linked GlcNAc transferase; OGA, O-GlcNAcase.

Glycosylation, an enzymatically-driven process initiating in the endoplasmic reticulum (ER) and maturing in the Golgi apparatus, represents a fundamental and ubiquitous protein modification with profound implications in lung cancer pathogenesis and clinical management (9) (**Figure 2**). Its functional diversity is exemplified through a complex repertoire of modifications: N-glycosylation of cathepsin V (at N221/N292) promotes lymph node metastasis and serves as a serum biomarker (10), while distinct N-glycan signatures on extracellular vesicles enable histological subtyping (11), and aberrant mucin O-glycosylation drives immune evasion (12). Beyond diagnostic utility, glycosylation actively regulates therapeutic response, as evidenced by tunicamycin chemosensitization through PTX3 modification and integrin β1 collaboration with glycosylated collagen in determining cancer stem cell fate (13, 14). Mechanistically, disease-specific haptoglobin beta chain N-glycosylation offers discriminative power while MS reveals elevated α1,6-/α1,2-/α1,3-linked fucosylation and sialylated fucosylated N-glycans mediating adhesion (15–17). Critically, O-GlcNAcylation opposes oncogene-induced senescence to promote transformation, whereas N-glycosylation appears to stabilize TIM-4 at Asn291 to enhance motility] and enables GPNMB-EGFR interaction at Asn134 to propel progression (18–20). Therapeutically, engineered sGal-3 exploits glycosylation for selective cytotoxicity, core-fucosylated SOD resists proliferation, and GALNT-mediated O-glycosylation of ITGA5 co-activates PI3K/AKT and MAPK/ERK pathways (21–23). Furthermore, O-GlcNAcylation​has been shown to stabilize SMAD4, thereby modulating TGF-β signaling (24), and ST6GalNAc-I-mediated sialylation dually orchestrates immune suppression (via NECTIN2-TIGIT) and matrix remodeling (via MUC5AC-VCAN-V1) (25). Collectively, these findings suggest glycosylation not as isolated events but as an interconnected regulatory network central to lung cancer malignancy, offering a compelling framework for novel diagnostic and therapeutic strategies.

## Phosphorylation and lung cancer

**Figure 3:**
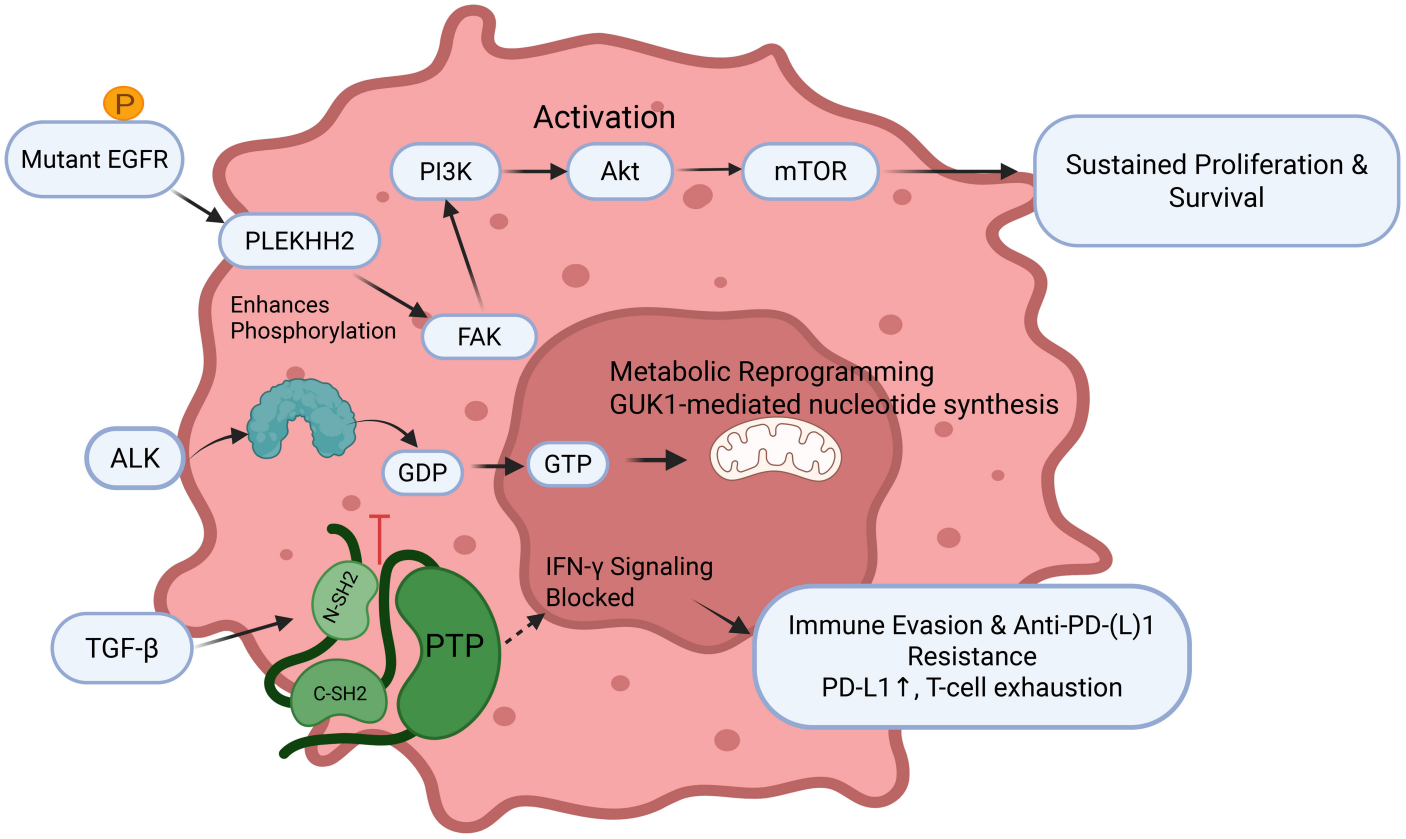
The central role of phosphorylation in driving lung cancer pathogenesis and therapeutic resistance​ EGFR, Epidermal Growth Factor Receptor; GDP, Guanosine diphosphate; GTP, Guanosine triphosphate; TGF-β, Transforming growth factor-β; FAK, Focal adhesion kinase.

As the most prevalent and evolutionarily conserved PTM, protein phosphorylation serves as the principal mechanism for cellular signal transduction across prokaryotic and eukaryotic organisms, functioning as a reversible covalent modification that centrally governs protein activity (**Figure 3**). This dynamic process involves kinases transferring phosphate groups to substrate proteins and phosphatases catalyzing their removal, collectively regulating lung cancer pathogenesis and therapeutic response. Mechanistically, diverse phosphorylation-mediated pathways contribute to oncogenesis: neurotensin receptor 1 (NTSR1) activation induces tyrosine phosphorylation of epidermal growth factor receptor (EGFR) and human epidermal growth factor receptor 3 (HER3), driving proliferation in non-small cell lung cancer (NSCLC) (26, 27), tobacco carcinogen NNK promotes liver kinase B1 (LKB1) hyperphosphorylation via β-adrenergic receptor/protein kinase A (β-AR/PKA) signaling (28), and hepatocyte nuclear PI3K/AKT activation to suppress malignant growth (29). Factor 1B (HNF1B)/protocadherin-α13 (PCDHα13) overexpression attenuates Chemoresistance emerges through JNK-mediated Bcl-2 phosphorylation impairing autophagy-dependent death (30), while curcumin restores apoptotic sensitivity via reactive oxygen species (ROS)-dependent p38 mitogen-activated protein kinase (MAPK) activation (31). Cell cycle regulation occurs through protein kinase C (PKC) substrate phosphorylation induced by bisindolylmaleimide derivatives (32), and pleckstrin homology domain-containing family H member 2 (PLEKHH2) enhances focal adhesion kinase (FAK) phosphorylation to activate PI3K/AKT signaling and promote invasion (33). Radiation resistance develops through kinesin light chain 2 (KLC2)-mediated reduction of p53 phosphorylation (34), while proliferation is driven by oncogenic EGFR-TNF receptor-associated factor 4 (TRAF4)-MAP kinase kinase kinase 3 (MEKK3)-extracellular signal-regulated kinase 5 (ERK5) axes (35). Therapeutic strategies include p53 reactivation through Ser392 phosphorylation targeting (36) and nickel chloride (NiCl_2_) promotes lung cancer invasion and metastasis by activating the IL-6/STAT3 pathway, which upregulates the E3 ligase TRIM31 to drive ubiquitination and degradation of the tumor suppressor TP53 (37), while chromium-induced carcinogenesis involves polo-like kinase 1 (PLK1) phosphorylation of pyruvate dehydrogenase E1 subunit alpha 1 (PDHA1) at Thr57, inducing mitochondrial dysfunction and mitophagy (38). Additional mechanisms include HORMA domain-containing protein 1 (HORMAD1)-mediated β-catenin stabilization through sequential AKT (Ser473) and glycogen synthase kinase-3β (GSK-3β) (Ser9) phosphorylation (39), zinc finger E-box binding homeobox 1 (ZEB1)-orchestrated PLK1-dependent kinetochore phosphorylation (40). RhoQ inhibition was found to enhance transforming growth factor-β (TGF-β)-Smad3 phosphorylation (41), tumor cells expressing SUR1 promote the transformation of normal fibroblasts into cancer-associated fibroblasts (CAFs) and tumor progression by reducing the delivery of tumor-suppressive let-7a-5p miRNA via exosomes (42), and long non-coding RNA LINC00473-mediated nuclear factor erythroid 2-related factor 2 (Nrf2) phosphorylation suppression inducing apoptosis (43). Immunologically, TGF-β blockade potentiates interferon-γ (IFN-γ) resistance to anti-programmed death ligand 1 (PD-L1) therapy by dysregulating Src homology region 2 domain-containing phosphatase 1 (SHP1) activity (44). This comprehensive phosphorylation network highlights promising diagnostic and therapeutic targets for lung cancer precision medicine.

## Ubiquitination and lung cancer

**Figure 4:**
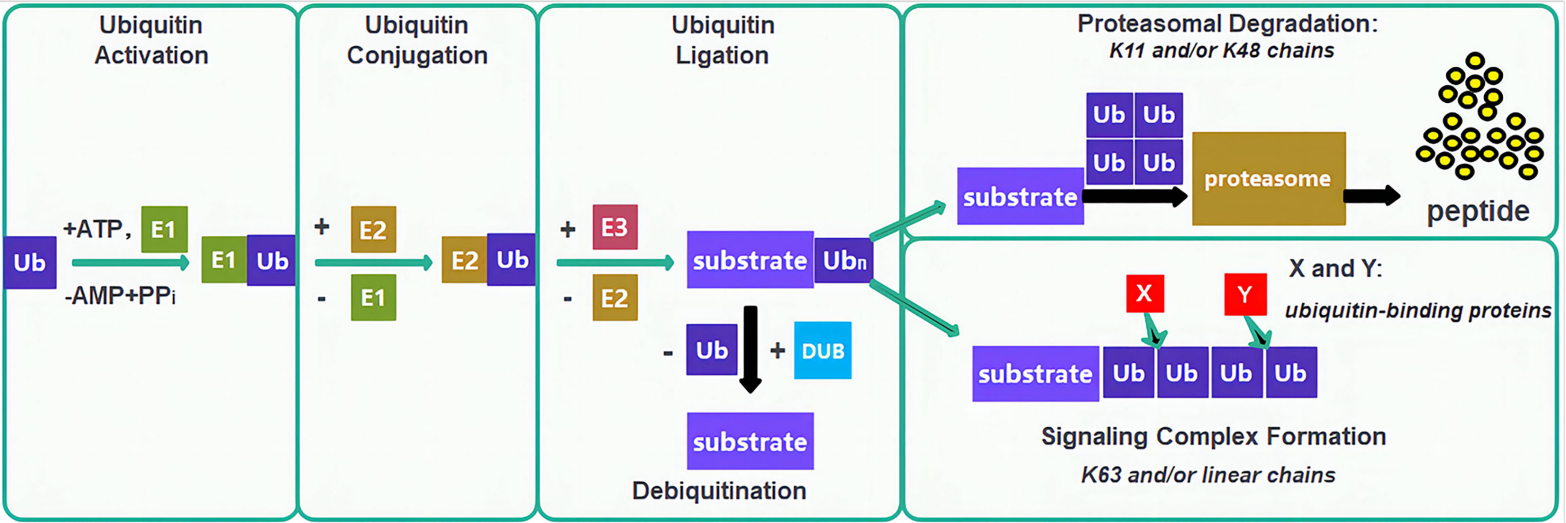
Ubiquitin-proteasome system and therapeutic targeting in lung cancer. The canonical E1-E2-E3 ubiquitination cascade; Ub, Ubiquitin; E1, Ubiquitin-activating enzymes; E2, Ubiquitin-conjugating enzymes; E3, Ubiquitin-protein ligases; DUB, Deubiquitinating enzyme.

Ubiquitination is a critical PTM process wherein ubiquitin molecules, under the orchestration of specialized enzymatic systems, including ubiquitin-activating enzymes (E1), ubiquitin-conjugating enzymes (E2), and ubiquitin-protein ligases (E3), covalently attach to target proteins to regulate their stability, activity, subcellular localization, and interactions, thereby playing a pivotal role in virtually all biological processes including immune regulation, mitophagy, DNA damage repair, cell cycle control, epigenetic modulation, proliferation, and apoptosis (45, 46)(**Figure 4**). In lung cancer, ubiquitination drives carcinogenesis through diverse mechanisms: the aPKC inhibitor DNDA promotes Cbl-b-mediated ubiquitination and degradation of PKC/FAK to suppress metastasis (47), UBE2O catalyzes degradation of Mxi1 to drive tumorigenesis (48), RNF-8 mediates K63-linked ubiquitination to activate Akt and promote chemoresistance (49), HECW1 catalyzes K48-linked polyubiquitination of Smad4 to facilitate NSCLC progression (50), UBE2T targets FOXO1 for degradation and activates Wnt/β-catenin signaling (51), FBW7 ubiquitinates Mcl-1 to inhibit anti-apoptotic signaling (52), USP51 stabilizes ZEB1 to confer cisplatin resistance (43). NEDD4L regulates CPNE1 degradation to modulate oncogenic signaling (54). MIB2 degrades Notch1 to exert tumor-suppressive effects (55). KLHL18 promotes p85α degradation to inhibit PI3K/AKT and PD-L1 signaling (56). Ablation of AdipoR4 enhances Keap1-mediated Nrf2 ubiquitination and degradation to increase chemosensitivity (57). SIRT3 modulates ubiquitin-dependent degradation of mutant p53 (58). TRIM2 catalyzes K48-linked ubiquitination of Snail1 to enhance invasion (59). lncRNA AFAP1-AS1 stabilizes c-Myc by inhibiting its ubiquitination to drive metastasis (60). USP37 deubiquitinates and stabilizes Snail to promote migration (61). circNDUFB2 facilitates ubiquitin-dependent degradation of IGF2BP1 and activates anti-tumor immunity (62). RNF152 catalyzes K48-linked ubiquitination of IRAK1 to downregulate AKR1B10 and suppress malignancy (63), and ROS-induced o8G modification of circPLCE1 enhances HSC70 ubiquitination to inhibit autophagy and tumor progression (64).

## Methylation and lung cancer

**Figure 5:**
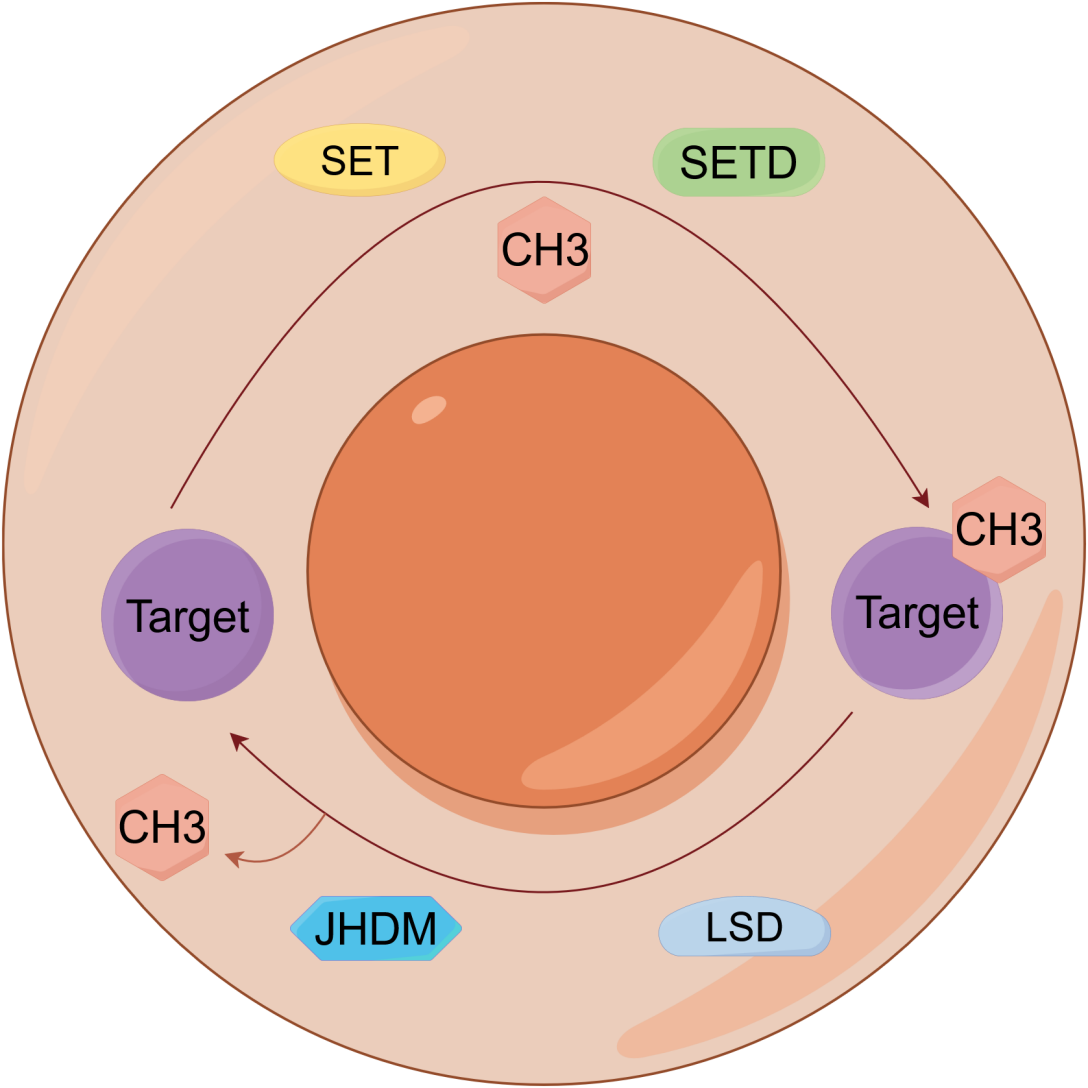
Epigenetic regulation by DNA and histone methylation in lung cancer. Dynamic regulation of epigenetic signaling through antagonistic protein methylation and demethylation on histone targets.​ SET, Su(var)3-9, Enhancer-of-zeste, Trithorax; SETD, SET Domain-containing; JHDM, Jumonji C-domain-containing histone demethylase; LSD, Lysine specific demethylase.

Methylation, a fundamental epigenetic and PTM, involves methyltransferases catalyzing the covalent transfer of methyl groups from S-adenosylmethionine (SAM) to specific residues on DNA, RNA, or proteins, dynamically regulating gene expression, protein function, and RNA metabolism (**Figure 5**) (65). Dysregulated methylation is mechanistically implicated in lung cancer pathogenesis, diagnosis, and therapy: DNA hypermethylation of tumor suppressors including RASSF1, ATIC, SHOX2 (mSHOX2) and PRKCDBP serves as a clinically validated biomarker for early detection, lymph node metastasis, and poor prognosis (66–68), promoter hypermethylation of SOX1 and SPAG6 contributes to transcriptional silencing and activates oncogenic pathways such as JAK/STAT (69, 70), non-coding RNAs including circTFF1 (via miR-29c-3p/DNMT3A/BCL6 axis), hsa_circ_0077837 (PTEN silencing), and HOXC-AS3 (activated by SETD1A-mediated H3K4me2) promote proliferation, invasion, and ferroptosis resistance (71–73). RNA methylation regulators METTL14 and METTL3 enhance stability and translation of oncogenes, such as PLAGL2 and DDX23, through m6A modification, activating β-catenin and PI3K/AKT pathways (74, 75). Histone methyltransferases SETD1A and SETDB1 catalyze H3K4 trimethylation and modulate SPG20 methylation to drive tumor progression and metastasis (76, 77), and the microRNA miR-26a-5p attenuates Wnt signaling by inhibiting DNMT3A-mediated SFRP1 promoter methylation (78). Additionally, YRNA fragment hY4F is secreted via methylated YBX1-packaged EVs, attenuating its tumor-suppressive function (79). Critically, NNMT upregulation depletes methyl donors, reducing global H3K9me3/H3K27me3 levels and establishing feedback loops via EGR1/ALDH3A1/lactate that sustain EGFR-TKI resistance, highlighting therapeutic targeting potential (80).

## Acetylation and lung cancer

**Figure 6:**
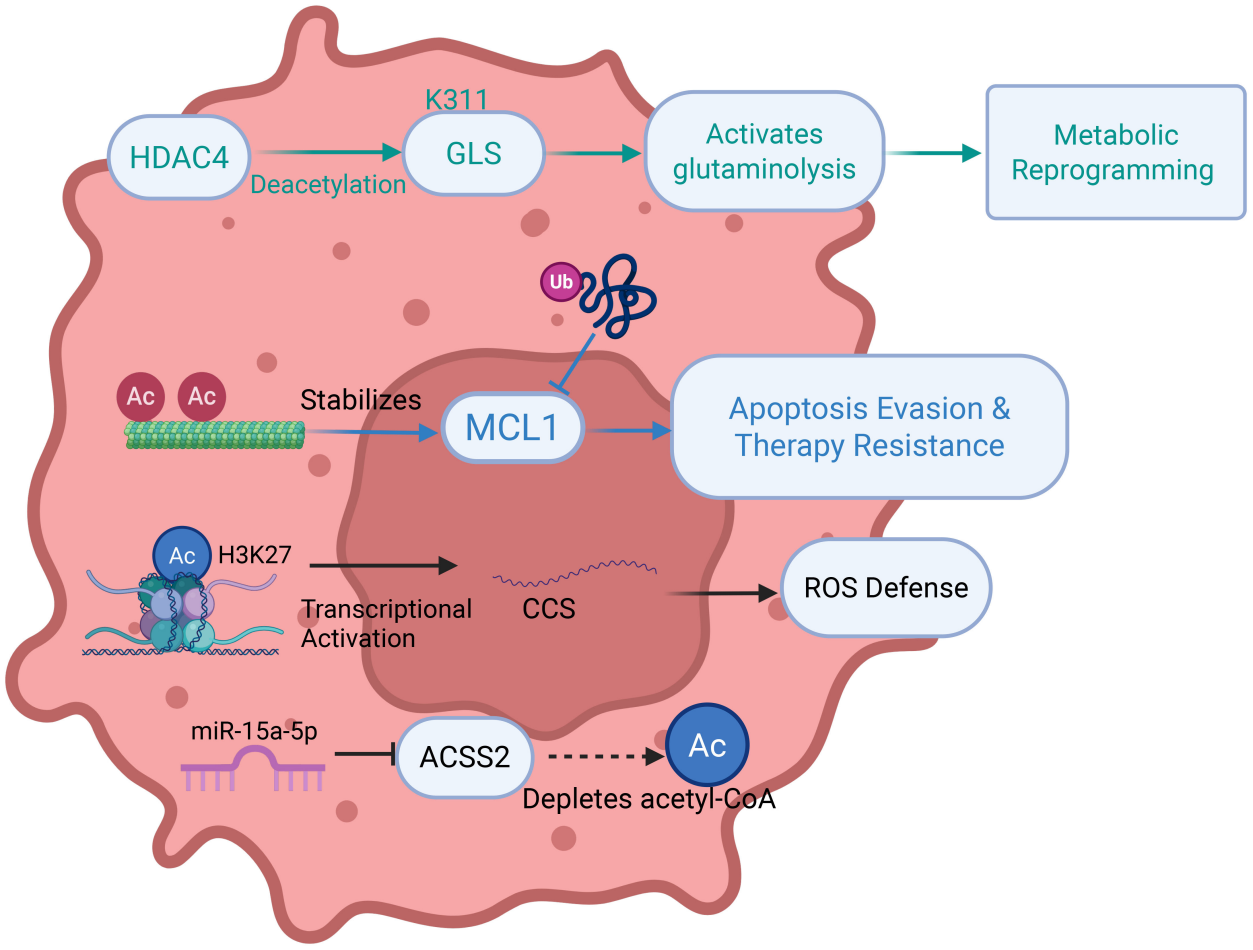
Mechanisms of acetylation in regulating metabolic reprogramming, cell death, and immune microenvironment in lung cancer. HDAC, Histone Deacetylase; GLS, Glutaminase; MCL1, Myeloidcell leukemia 1;​ AC, Acetyl group; CCS, Copper Chaperone for Superoxide dismutase; ACSS2, Acyl-CoA Synthetase Short-Chain Family Member 2.

Acetylation, a reversible PTM catalyzed by acetyltransferases (HATs/KATs), involves the transfer of acetyl groups to lysine ϵ-amino groups, dynamically regulating protein function, chromatin architecture, and gene expression by modulating histone tail modifications and transcription factor accessibility (**Figure 6**) (81). This versatile modification orchestrates cellular processes through multiple mechanisms: modulating DNA-protein interactions via histone acetylation, fine-tuning protein interactions via non-histone protein conformational changes, allosterically regulating enzymatic activity, and directing subcellular localization through nuclear localization signal masking (82, 83). In lung cancer, acetylation plays pivotal roles in pathogenesis and therapy: miR-15a-5p targets ACSS2 to inhibit acetyl-CoA production, suppressing lipid metabolism and histone acetylation-mediated transcription to attenuate metastasis (84). Acetylated α-tubulin stabilizes MCL1 by shielding it from ubiquitin ligase recognition and inhibiting K48-linked polyubiquitination, determining paclitaxel sensitivity (85). CBP/p300-mediated acetylation of KPNA2 promotes its nuclear export and attenuates oncogenicity (86). HDAC4 deacetylates GLS at K311 to activate glutaminolysis, while H3K27 acetylation epigenetically​is correlated with the activation of CCS transcription, enhancing ROS scavenging and cytoprotective autophagy (87, 88). Suberoylanilide hydroxamic acid (SAHA) induces radiosensitization in lung cancer cells by promoting K120 acetylation of p53, which regulates mitochondrial apoptosis, and this effect requires specific p53 status (89). AKR1C1 acetylation at K185/K201 enhances enzymatic activity and metastatic potential (90). Shikonin inhibits c-Myc-mediated HDAC1 recruitment to ATF3, promoting local histone acetylation (91). SIRT5 promotes the progression of non-small cell lung cancer by inducing deacetylation and reducing the expression of FABP4 (92). Additionally, ACAT1-mediated hypersuccinylation elevates ROS and impedes tertiary lymphoid structure formation, promoting anti-PD1 resistance, highlighting acetylation-related pathways as promising therapeutic targets (93).

## Succinylation, crotonylation and lung cancer

**Figure 7:**
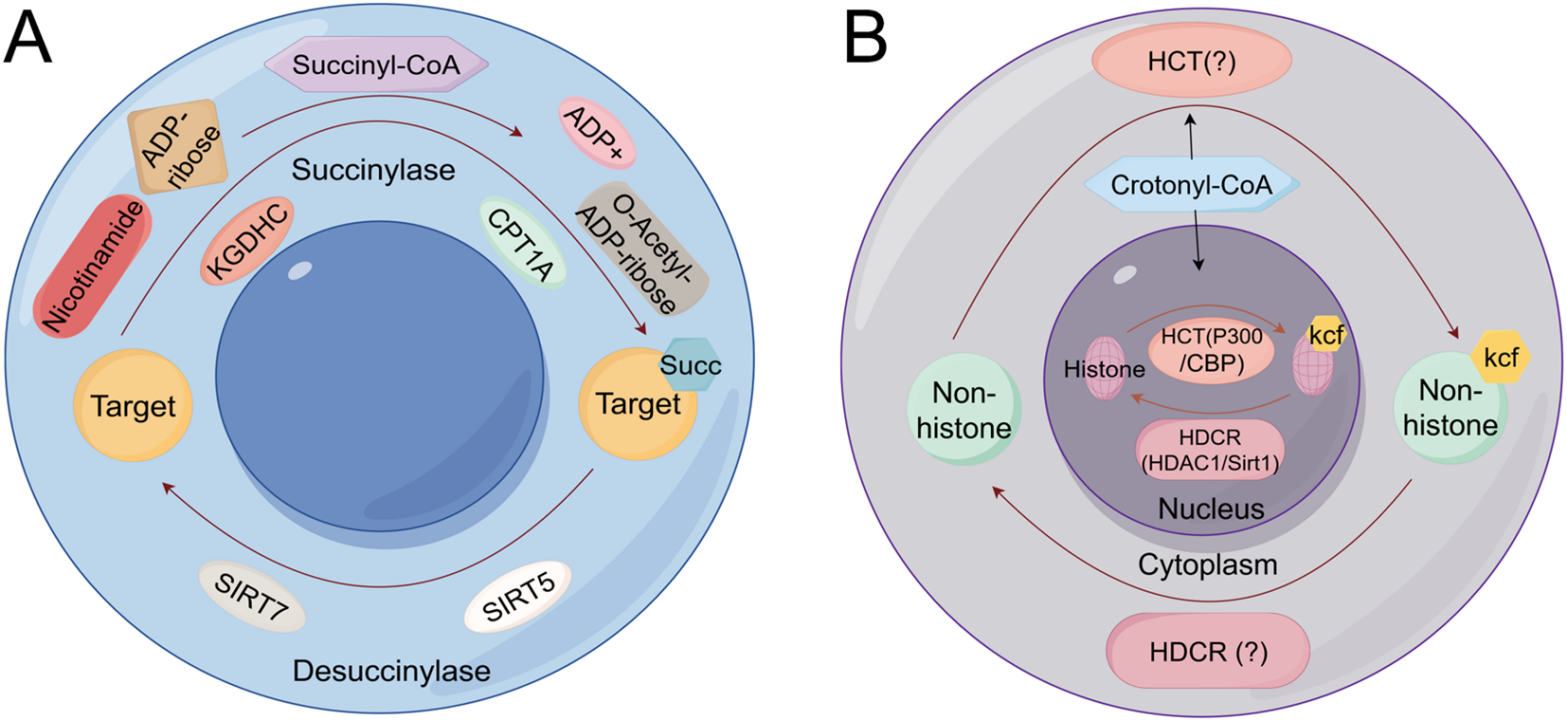
Succinylation and crotonylation mechanism schematic diagram **(A)** The mitochondrial succinylation-desuccinylation cycle **(B)** The nuclear crotonylation-decrotonylation cycle catalyzed by HCTs and HDCRs. KGDHC, Ketoglutarate Dehydrogenase Complex; ADP, Adenosine Diphosphate; SIRT5, Sirtuin 5; SIRT7, Sirtuin 7; HDCR, Histone Decrotonylase; HCT, Histone ​Crotonyltransferase.​.

Protein succinylation and crotonylation represent emerging and critically important PTMs that significantly contribute to lung cancer pathogenesis by dysregulating metabolic signaling and cell death pathways (**Figure 7A**). Succinylation involves the enzymatic transfer of a succinyl group (-CO-CH2-CH2-COO-) from succinyl-CoA to lysine ϵ-amino groups, playing key regulatory roles in the tricarboxylic acid (TCA) cycle, amino acid metabolism, and fatty acid metabolism (94), whereas crotonylation entails crotonyl-CoA-mediated transfer of crotonyl groups to histone residues, crucially influencing gene expression and other biological processes (**Figure 7B**) (95). Specifically, succinylation of superoxide dismutase 1 (SOD1) diminishes its enzymatic activity, and mutation at the SOD1 succinylation site suppresses lung tumor growth, underscoring its therapeutic potential (96). Additionally, succinylation at lysine 93 (K93) stabilizes succinate-CoA ligase subunit beta (SUCLG2), enhancing its abundance and promoting LUAD proliferation (97). Furthermore, crotonylation of brain expressed X-linked 2 (BEX2) promotes its interaction with nuclear dot protein 52 (NDP52), augmenting mitophagy and attenuating chemotherapy-induced apoptosis (98). Collectively, these findings highlight the intricate and multifaceted roles of succinylation and crotonylation in lung cancer, revealing novel mechanistic insights into metabolic reprogramming, drug response, and survival pathways, thereby offering promising targets for therapeutic intervention.

## SUMOylation and lung cancer

**Figure 8:**
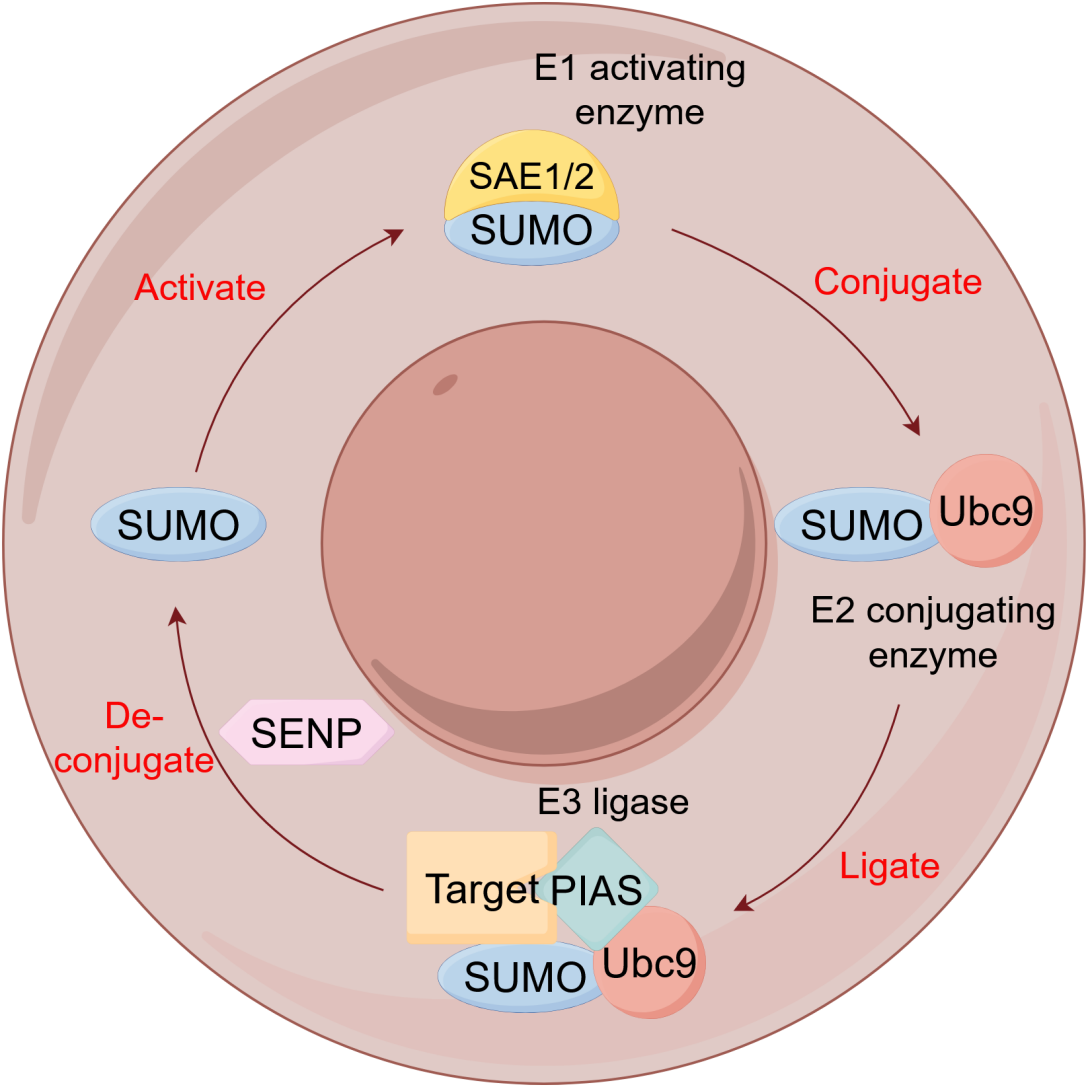
The SUMOylation cycle. SUMO, Small Ubiquitin-like modifier; SAE, SUMO-activating enzyme; Ubc9, Ubiquitin-conjugating enzyme 9; PIAS, Protein Inhibitor of Activated STAT; SENP, SUMO/sentrin-specific protease.

SUMOylation, the covalent attachment of small ubiquitin-related modifier (SUMO) isoforms (SUMO1-4) to target proteins, represents a highly conserved and critical PTM that regulates diverse cellular processes including cell cycle progression, nuclear transcription, protein interactions, DNA damage repair, and differentiation (**Figure 8**) (99). In lung cancer, SUMOylation contributes centrally to pathogenesis and therapy resistance through multiple mechanisms: Ubc9/PIASy-mediated SUMOylation of Slug promotes NSCLC metastasis by enhancing its transcriptional repression activity through HDAC1 recruitment (100), PIAS1 facilitates SUMO1-SMAD4 complex formation, enhancing vimentin expression and cell migration (101). Hypoxia promotes epithelial-mesenchymal transition and lung cancer metastasis by downregulating SIRT1 expression in a SUMOylation-dependent manner (102). HSP70 promotes HIF-1α SUMOylation under hypoxia, conferring ferroptosis resistance and tumor recurrence after ablation (103). KEAP1 SUMOylation modulates ROS production and disrupts KEAP1-NRF2 interaction to activate antioxidant responses (104). VEGFR2 SUMOylation attenuates downstream signaling, suppressing proliferation, migration, and angiogenesis (105). HSP70 upregulates SUMO1-mediated HIF-1α modification to enhance thermotolerance and induce aberrant immune responses (106). PIASy enhances GATA2 SUMOylation, reducing its transcriptional activity (107). RNF4 enhances the tumor-suppressive function of NDRG2 in lung adenocarcinoma by promoting its SUMOylation (108), and SUMOylation-mediated activation of ALIX upregulates extracellular vesicle-derived circTLCD4-RWDD3 to promote lymphatic metastasis (109). Furthermore, a SUMOylation-related prognostic signature for osimertinib resistance has been identified, with gene expression correlating with immune activation and offering novel biomarkers and therapeutic targets (110).

## PTM-based diagnostic and prognostic tools in lung cancer​​

PTMs have emerged as powerful biomarkers for lung cancer detection, stratification, and therapeutic response prediction. Phosphoproteomic profiling of EGFR-mutant lung adenocarcinoma identifies specific phosphorylation events, such as EGFR-pTyr1197, MAPK7-pTyr221, and DAPP1-pTyr139, that serve as quantitative biomarkers of TKI sensitivity, with their inhibition dynamics directly correlating with drug response, offering real-time pharmacodynamic insights (111). Beyond phosphorylation, glycosylation patterns also show clinical relevance: Zhang et al. developed a machine learning-based PTM Learning Signature (PTMLS) from multi-cohort analysis of 1,231 LUAD cases, identifying beta-1,4-galactosyltransferase 2 (B4GALT2) as a key prognostic biomarker within this framework, where elevated B4GALT2 expression correlates with poor survival and CD8^+^ T-cell exclusion, suggesting an immunoevasive role in LUAD progression (112). Epigenetic modifications further expand the biomarker landscape: EZH2, the catalytic subunit of the PRC2 complex, mediates H3K27 trimethylation (H3K27me3) to drive lung carcinogenesis, and integrated analysis of H3K27me3-nucleosome levels with ctDNA profiling significantly enhances diagnostic accuracy. Notably, elevated H3K27me3 is detected in 25.5% of treatment-naïve patients lacking somatic mutations, and this epigenetic signature improves the detection rate of disease progression from 43.1% to 58.2%, underscoring its dual utility for non-invasive diagnosis and molecular residual disease (MRD) monitoring in lung cancer (113). Supporting the role of glycosylation in immune regulation, a foundational study suggested that glycosylation of PD-L1 stabilizes its expression and inhibits T-cell function, a mechanism that underpins the efficacy of immune checkpoint inhibitors (114). Histone methylation modifications further expand the epigenetic biomarker landscape. EZH2-mediated H3K27 trimethylation (H3K27me3) drives lung carcinogenesis. While direct detection of H3K27me3 in ctDNA is emerging, the analysis of cancer-specific DNA methylationpatterns in ctDNA has become the gold standard for molecular residual disease monitoring (115). The prospective study NCT06358222 demonstrated that combining ctDNA methylation profiling with PET-CT imaging can accurately predict lymph node metastasis in non-small cell lung cancer preoperatively (116). Acetylation markers are also gaining traction as predictors of therapy response, high HDAC1 expression is significantly associated with poor lung cancer differentiation, squamous cell carcinoma subtype, and unfavorable patient prognosis, suggesting its potential as a diagnostic and prognostic marker (117).

Collectively, these findings highlight the multidimensional value of PTM-based biomarkers.​The ongoing integration of multi-level PTM profiling, from phosphoproteomics to epigenomics, into large-scale clinical trials promises to further refine lung cancer subclassification, monitor therapeutic efficacy, and guide personalized treatment strategies.

## Therapeutic targeting of PTM pathways​

Studies showed that the combination of Rapamycin and SAHA enhances radiosensitivity in non-small cell lung cancer by inducing acetylation and autophagy, thereby inhibiting DNA damage repair (118). The combination of vorinostat and cisplatin significantly enhances antitumor efficacy against small cell lung cancer *in vitro* and *in vivo* by increasing histone acetylation levels and suppressing thymidylate synthase expression (119). Plant homologous structural domain finger protein 23 (PHF23) promotes tumor proliferation and migration, yet enhances cisplatin/docetaxel sensitivity by facilitating DNA damage repair. PHF23 stabilizes ACTN4 through its PHD domain by inhibiting K48-linked ubiquitination, implicating it as a therapeutic target (120). Emerging therapeutic strategies employ PROTAC-mediated ubiquitination to degrade KRASG12C mutant proteins, offering a viable approach to reduce oncogenic KRAS levels and suppress downstream signaling pathways in cancer cells (121). While when NSCLC cells were treated with norcantharidin (NCTD), a demethylated form of cantharidin, a reduction in both the mRNA and protein levels of YAP, as well as increased YAP phosphorylation were observed, which inhibited the proliferation of NSCLC cells (122). PRMT6 promotes glycolysis and cisplatin resistance through methylation of metabolic enzymes 6PGD (R324) and ENO1 (R9/R372). The PRMT6 inhibitor DCPR049_12 effectively reverses these effects and enhances chemosensitivity (123).

## Clinical translation of PTMs: diagnostic and therapeutic decision-making​

The intricate landscape of PTMs in lung cancer presents both challenges and opportunities for clinical translation (4, 5, 8). We will organize the most promising PTM-related findings around core clinical issues, such as diagnosis, predictive biomarkers, and therapeutic targeting, with the aim of clarifying the role of PTM research advancements in these critical areas (**Table 2**).

**Table 2:** Clinically actionable PTM biomarkers and targets in lung cancer.

Clinical application	PTM type	Key molecule	Clinical implication	Associated lung cancer	Reference
Diagnosis and Early Detection	Glycosylation	Glycosylated cathepsin V	Biomarker for lymph node metastasis	NSCLC	(10)
		Extracellular Vesicle N-glycans	Histological subtyping of lung cancer.	NSCLC and SCLC	(11)
		Haptoglobin beta chain	Distinct signatures of lung cancer types	LUAD and LUSC	(12)
	Phosphorylation	​​LKB1	Pathogenesis Markers	NSCLC	(28)
		PLEKHH2	Cell proliferation, migration, invasion	NSCLC	(33)
		HORMAD1	Prognostic biomarker	LUAD and LUSC	(39)
	Ubiquitination	HECW1	Prognostic marker	NSCLC	(50)
		Notch1, Snail1/TRIM2, c-Myc​	Invasion and metastasis prediction	NSCLC	(55, 59, 60)
	Methylation	SHOX2, RASSF1A, PRKCDBP	Early detection of lung cancer	NSCLC	(66–68)
		SOX1, SPAG6	Diagnostic signal	NSCLC	(69, 70)
	Acetylation	​​FABP4​	Promotes progression	NSCLC	(92)
	SUMOylation	​SMAD4	Prediction of metastasis and invasion	NSCLC	(101)
		VEGFR2	Prognostic marker	NSCLC	(105)
Therapy Response and Therapeutic Targeting	Glycosylation	PTX3	Tunicamycin-induced inhibition of glycosylation can chemosensitize tumors by modifying PTX3	NSCLC	(13)
		TIM-4, GPNMB, SMAD4	Promote progression and therapy resistance	NSCLC	(19, 20, 24)
		GALT2, ST6GalNAc-I	Proliferation, migration, and invasion	NSCLC, LUAD	(23, 25)
	Phosphorylation	Bcl-2, KLC2​	Chemoresistance, radiation resistance	NSCLC	(30, 34)
		p53	​​Therapeutic Targeting	NSCLC	(36)
		​TGF-β/SHP1	​​Immunotherapy Resistance	LUAD mouse model	(44)
	Ubiquitination	UBE2O	radiosensitization target	NSCLC	(48)
		RNF-8	Cell proliferation and resistance to chemotherapy	NSCLC	(49)
		FBW7/Mcl-1	Epigenetic therapy	NSCLC	(52)
		​​ZEB1	Therapeutic target for DDP resistance	NSCLC	(53)
	Methylation	hsa_circ_0077837	Promoting the epigenetic silencing of the tumor suppressor PTEN	NSCLC	(73)
		METTL3, METTL14	Contributing to therapy resistance, Potential therapeutic targets	NSCLC	(74, 75)
		SETD1A, SETDB1​​	Suppress tumor metastasis, potential epigenetic drug targets	NSCLC	(76, 77)
	Acetylation	CCS, AKR1C1	Therapeutic target	LUAD	(88, 90)
		​​MCL1​​	Chemotherapy Sensitivity	NSCLC	(85)
		​​SAHA	Radiosensitization	NSCLC	(89)
		​​Shikonin/c-Myc/HDAC1/ATF3, ACAT1	Immunotherapeutic target	NSCLC	(91, 93)
	Succinylation	SUCLG2, BEX2/NDP52	Therapeutic target	LUAD, NSCLC	(97, 98)
	SUMOylation	Slug, KEAP1-NRF2	Therapeutic targets	NSCLC	(100, 104)
		HIF-1α	Inducing recurrence	NSCLC	(103)

The growing adoption of liquid biopsies has accelerated the search for non-invasive diagnostic biomarkers, and PTMs, owing to their stability and mechanistic relevance to tumor biology, which represent ideal candidates. DNA methylation-based assays, such as the detection of SHOX2 and RASSF1 hypermethylation in plasma ctDNA, currently lead in clinical translation (66, 67), highlighting the validated clinical utility of this PTM type. Beyond DNA modifications, emerging epigenetic and glycomic signatures are showing strong diagnostic potential: for example, H3K27me3-modified nucleosomes in plasma can enhance the sensitivity of ctDNA-based minimal residual disease monitoring (113), while MS-based profiling of serum protein glycosylation patterns, such as sialylation and fucosylation exhibiting high discriminatory power for early-stage detection, suggesting promise for future multi-analyte liquid biopsy panels (16, 25).

The intricate interplay between genomic alterations, dynamic PTM networks, and therapeutic response necessitates a structured framework to guide clinical decision-making in lung cancer. For instance, in EGFR-mutant lung adenocarcinoma, quantitative assessment of EGFR-pTyr1197 phosphorylation via targeted MS of liquid biopsy samples or immunohistochemistry on serial tumor biopsies provides a direct pharmacodynamic readout. A rapid decline in phosphorylation signals effective inhibition and predicts favorable response to tyrosine kinase inhibitors, whereas its rebound heralds acquired resistance, prompting a switch to combination therapies, such as adding a SRC inhibitor upon detecting SRC kinase activation in ALK-positive patients (111, 124). Beyond phosphorylation, glycosylation remodeling offers critical insights into the immune microenvironment. IHC-based detection of B4GALT2 overexpression in tumor tissue identifies patients with CD8^+^ T-cell excluded phenotypes, who are less likely to respond to immunotherapy (112). Similarly, PD-L1 glycosylation, detectable by IHC or emerging serum MS/ELISA platforms, stabilizes PD-L1 and inhibits T-cell function, underscoring its role as a resistance mechanism to immune checkpoint blockade (114). The feasibility of PTM monitoring is increasingly supported by advancing technologies. Liquid biopsy-based methylation-specific PCR or MS for *SHOX2/RASSF1A* in ctDNA is already a clinically validated tool for disease detection and MRD monitoring (66, 67, 125). Similarly, immunoaffinity enrichment coupled with MS allows for the detection of H3K27me3-modified nucleosomesin plasma, significantly enhancing the sensitivity of progression detection when combined with ctDNA mutation analysis (113). The integration of dynamic PTM profiling into the clinical workflow, marks a paradigm shift towards a more responsive and precise form of oncology care (**Table 3**).

**Table 3:** PTM-guided precision therapy for lung cancer.

PTM type	Dynamic PTM layer	Sample	Clinical implication and therapeutic utility	Reference
DNA Methylation (SHOX2, RASSF1A)	Gene Silencing/Activation	Plasma ctDNA	Biomarker for disease detection and MRD monitoring	(66, 67, 125)
Histone Modification​ (H3K27me3)	Chromatin State/Gene Expression	Plasma Nucleosomes	Enhances sensitivity of ctDNA-based progression detection	(113)
Phosphorylation (EGFR-pTyr1197)	Kinase Activity/Signaling Pathway	Liquid Biopsy or Tumor Biopsie	Predict TKI sensitivity or prompt a switch to combination therapy	(111, 124)
Glycosylation (Serum Proteins)​​	Protein Function/Cell Interaction	Serum	A promising diagnostic biomarker for future multi-analyte liquid biopsy panels	(16, 25)
Glycosylation (B4GALT2 Overexpression)	Tumor Microenvironment	Tumor Tissue	Identifies CD8^+^ T-cell excluded phenotype, predicting poor response to immunotherapy	(112)
Glycosylation(PD-L1)	Immune Checkpoint Function	Tumor Tissue or Serum	Indicates the need for combination therapy strategies	(114)

Furthermore, we systematically assessed the validation status of the major PTM-based findings discussed herein (**Table 4**). The applications discussed in this review are therefore organized according to their current level of clinical validation, which spans from clinically implemented assays, such as detection of *SHOX2/RASSF1A* methylation in liquid biopsies, and biomarkers correlated with outcomes in clinical cohorts, such as Trim35-mediated ubiquitination of LSD1 as a predictor of immunotherapy response, to promising preclinical findings, such as B4GALT2 glycosylation promoting immune exclusion, and novel mechanistic insights, such as the role of histone lactylation that await further investigation. This framework also documents specific limitations, including single-center studies and small sample sizes, aiming to provide a clear benchmark for assessing the maturity of each PTM-driven strategy.

**Table 4:** Major PTM-based findings categorized by level of clinical validation.

PTM type	Biomarker	Proposed clinical application	Level of evidence	Reference
DNA Methylation	SHOX2/RASSF1A	Diagnosis and early detection	IVD Approved​(Epi proLung^®^ assay). Validated in multicenter cohorts	(66, 67)
Phosphorylation	EGFR-pTyr1197	Pharmacodynamic monitoring of TKI response	Preclinical (Strong cell/animal data, correlation in human tissue samples)	(111)
​Glycosylation	B4GALT2 overexpression	Biomarker for CD8+ T-cell excluded phenotype and predictor of poor response to immunotherapy​	​​Clinical cohort (Validated in multi-center retrospective cohorts (n=1231)	(112)
​Ubiquitination​	Trim35 expression level​	Predictive biomarker for immunotherapy response and LSD1-targeted therapy in NSCLC​	Clinical Translational: comprehensive mechanistic discovery with robust multi-center clinical validation and direct therapeutic implications	(126)
Methylation​	PRMT6 overexpression​​	Predictive biomarker for cisplatin resistance and therapeutic target for combination therapy	Preclinical-Clinical Translational: mechanistic data in cell and animal models with correlation in human cohorts	(123)
Acetylation​	H1.4K75ac	Prognostic biomarker, potential therapeutic target	Mechanistic data in cell and animal models	(127)
SUMOylation​	SUMOylated SMAD4	​Therapeutic target for inhibiting NSCLC metastasis	Preclinical: mechanistic data in cell models	(101)

## Conclusion and outlook

The intricate involvement of PTMs in lung cancer pathogenesis has unveiled a wealth of novel therapeutic targets and strategies. Targeting the ubiquitin-proteasome system has yielded significant clinical advances. For instance, the E3 ligase Trim35 suppresses LSD1 demethylase activity via K63-linked polyubiquitination at Lys422, serving as a predictive biomarker for immunotherapy response in NSCLC (114). Furthermore, plant homeodomain (PHD) finger protein 23 (PHF23) stabilizes ACTN4 by inhibiting its K48-linked ubiquitination, promoting tumor progression yet paradoxically enhancing cisplatin/docetaxel sensitivity by facilitating DNA damage repair, presenting a complex but exploitable therapeutic node (115).

Beyond ubiquitination, inhibiting specific modifying enzymes represents a mainstream approach. Treatment with norcantharidin (NCTD) reduces YAP expression and promotes its inactivation phosphorylation, effectively inhibiting NSCLC proliferation (128). This aligns with the development of TEAD palmitoylation inhibitors that target the downstream Hippo pathway effector, currently in phase I/II trials for NSCLC (129).Similarly, protein arginine methyltransferase 6 (PRMT6) promotes glycolysis and cisplatin resistance by methylating metabolic enzymes 6PGD and ENO1. The PRMT6 inhibitor DCPR049_12 effectively reverses these effects and enhances chemosensitivity (123). The clinical potential of PRMT inhibition is underscored by the ongoing evaluation of PRMT5 inhibitors in solid tumors (NCT02783300), highlighting the druggability of this enzyme class (130).

Combination therapies targeting PTM-mediated resistance mechanisms are increasingly vital. In ALK-positive NSCLC, phosphorylation-mediated activation of SRC kinase contributes to drug resistance, and combined ALK/SRC inhibition significantly improves therapeutic efficacy (131). Chen et al. identified acetylation of histone H1.4 at K75 as a novel oncogenic mechanism, with the H1.4K75 mutation suppressing malignancy, presenting a compelling rationale for developing inhibitors targeting this acetylation site (127). The clinical success of Valemetostat, a novel dual inhibitor of EZH1 and EZH2, suggested significant clinical efficacy with a 44% objective response rate in a phase 2 trial involving 119 patients with relapsed or refractory peripheral T-cell lymphoma, establishing EZH inhibition as a promising therapeutic strategy for T-cell malignancies (132).

It is worth noting that some studies highlight the emerging significance of cross-talk among PTMs as a pivotal mechanism underlying therapy resistance in lung cancer. Kim et al. (24) revealed that O-GlcNAcylation at Thr63 of SMAD4 impedes its interaction with GSK-3β, thereby suppressing ubiquitin-mediated degradation and stabilizing SMAD4 to enhance TGF-β signaling, which promotes epithelial-mesenchymal transition and metastasis. Complementing this, Wattanathamsan et al. (85) suggested that tubulin acetylation, induced by chemotherapeutic stress, recruits and stabilizes the anti-apoptotic protein Mcl-1 on microtubules, inhibiting its polyubiquitination and conferring resistance to paclitaxel-induced apoptosis. Further expanding this paradigm, Peng et al. (103) showed that HSP70-mediated SUMOylation of HIF-1α after insufficient radiofrequency ablation not only drives tumor recurrence but also suppresses ferroptosis by downregulating key effectors, such as SLC7A11 and ACSL3. Collectively, these studies underscore a synergistic cross-talk between O-GlcNAcylation, acetylation, and SUMOylation pathways, which converge to stabilize oncogenic proteins, such as SMAD4, Mcl-1, HIF-1α, and coordinately inhibit apoptosis. This mechanistic insight advocates for targeting PTM networks as a promising strategy to overcome resistance.

PTMs play a significant role in cell growth, cell signaling regulation, protein localization, and maintaining cellular function by altering protein structure and function. The study of the mechanisms and functions of protein PTMs offers new opportunities in biopharmaceuticals, promising more precise and effective diagnostics and treatments. It can also provide new targets and screening methods for drug discovery and development, potentially accelerating the discovery and development of new drugs.

## Limitations and future perspectives

While this review has synthesized the critical roles of diverse PTMs, including phosphorylation, ubiquitination, methylation, acetylation, and SUMOylation, in driving lung cancer pathogenesis and therapy resistance, a fundamental limitation inherent to our synthesis, and to much of the current literature, is its reliance on a static and compartmentalized analytical framework. The majority of evidence discussed herein is derived from single-timepoint analyses of cell lines or clinical samples, captured either at baseline or upon disease progression. This approach, while invaluable for establishing mechanistic links, fails to capture the dynamic evolution and intricate crosstalk of PTM networks throughout the entire therapeutic continuum. To bridge this gap and truly translate PTM biology into clinically actionable strategies, future research must pivot towards longitudinal PTM monitoring, such as the integration of serial liquid biopsy protocols into prospective clinical trial designs.​Implementing such a strategy would move the field beyond a static snapshot to a dynamic movie of tumor evolution, which has extremely positive implications for the development of predictive biomarkers and the prevention and treatment of tumors.

The clinical translation of PTM-based biomarkers faces significant technical hurdles across primary detection methodologies. A foremost limitation is the inherent lack of antibody specificity for modified epitopes in immunoassays, where cross-reactivity and sensitivity to adjacent modifications risk yielding false-positive interpretations of PTM abundance and signaling activity. Furthermore, quantitative MS, despite its unbiased discovery power, grapples with substantial technical variability in sample preparation and instrument performance, necessitating robust yet challenging normalization strategies to ensure accurate quantification of low-abundance PTM peptides. Compounding these issues, pre-analytical variability in extracellular vesicle (EV) isolation, driven by methodological differences and sample handling protocols, introduces profound inconsistencies in yield, purity, and detected PTM signatures, severely limiting the reproducibility of liquid biopsy-based approaches. Addressing these multifaceted barriers demands a concerted effort to develop highly specific detection reagents, establish standardized proteomic workflows, and implement international consensus protocols for EV analysis, which are essential prerequisites to realizing the full clinical potential of dynamic PTM profiling.
